# Pragmatic analysis of the electric submerged arc furnace continuum

**DOI:** 10.1098/rsos.170313

**Published:** 2017-09-06

**Authors:** K. Karalis, N. Karkalos, G. S. E. Antipas, A. Xenidis

**Affiliations:** School of Mining Engineering and Metallurgy, National Technical University of Athens, Zografou Campus, Athens 15780, Greece

**Keywords:** electric arc furnace, slag, ferronickel, magnetohydrodynamics, properties, bubbles

## Abstract

A transient mathematical model was developed for the description of fluid flow, heat transfer and electromagnetic phenomena involved in the production of ferronickel in electric arc furnaces. The key operating variables considered were the thermal and electrical conductivity of the slag and the shape, immersion depth and applied electric potential of the electrodes. It was established that the principal stimuli of the velocities in the slag bath were the electric potential and immersion depth of the electrodes and the thermal and electrical conductivities of the slag. Additionally, it was determined that, under the set of operating conditions examined, the maximum slag temperature ranged between 1756 and 1825 K, which is in accordance with industrial measurements. Moreover, it was affirmed that contributions to slag stirring due to Lorentz forces and momentum forces due to the release of carbon monoxide bubbles from the electrode surface were negligible.

## 1. Introduction

Nickel is of particular economic consequence in the production of stainless steels, superalloys and fuel cells [1,2]. The principal nickel production route comprises the reductive smelting of calcine in electric submerged arc furnaces (EAFs); calcine is the yield of reductive roasting of nickelferrous lateritic ores in rotary kiln furnaces [3–5].

The majority of industrial smelting installations employ alternating current EAFs, in which the electric current required to maintain smelting is introduced via a number of self-baking graphite (Söderberg) electrodes. Owing to the poor electric conductivity of the calcine, the electric energy leads to the formation of a molten slag layer due to the effect of Joule heating and, to a lesser extent, because of the development of multiple small-scale electric arcs formed in the vicinity of the electrodes [6]. The shape of the electrode tip (most frequently rectangular or ellipsoidal) and the immersion depth of the electrodes affect slag transport properties—principally viscosity, thermal and electrical conductivity [7–9]. In turn, transport properties affect the maximum temperatures achieved, the temporal distribution of liquid fraction and the formation of stirring velocity gradients in the slag melt. Buoyancy effects are the main contributors to slag stirring [7]; however, marginal contributions also exist, due to electromagnetic (Lorentz) forces and rising CO bubbles emitted from electrode surfaces upon their reduction by oxygen present in the slag mixed-oxide phases [6,7,10–13].

In view of the physical complexity involved in reductive smelting, analytically descriptive efforts tend to separate fluid flow from electromagnetic phenomena [6,12,14–17]; hence, here we attempt the coupling of fluid flow, electromagnetic phenomena, melting and discrete phase phenomena (e.g. the interaction of CO bubbles with the bath), as a rational progression step towards a pragmatic description of the EAF continuum.

## 2. Model formulation

We set out to provide a theoretical account of the effect of the thermal and electrical conductivity and the viscosity of the slag on the liquid fraction and velocity profile in the slag region via transient analysis. Our approach caters for the effect of mixing due to the formation of CO bubbles as well as due to the presence of an external magnetic field.

**Figure 1.**
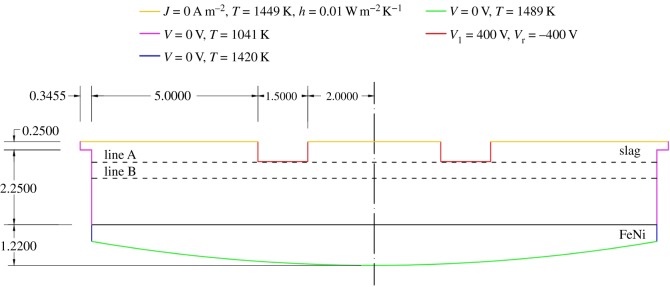
Dimensions, boundary conditions and phases inside the EAF.

Figure 1 is a schematic of the EAF in the industrial complex of LARCO S.A. in Larymna, Greece, considered in the current study. In our transient formulation we ignored the firebricks region, as the latter would substantially increase the number of nodal equations required to be solved, thus increasing computational expense. Additionally, due to its low viscosity, the air region was omitted from the model, as it was considered to not significantly affect the outcome of the simulations. Both the firebricks and air regions were replaced by appropriate boundary conditions, as described in forthcoming §2.2. Additionally, our approach accounted for the effect of both rectangular and ellipsoidal electrode tip geometries. For each tip, immersion depths of 0.4, 0.6 and 0.8 m were considered, to which electric potential values of 100, 200, 300 and 400 V were applied.

### 2.1. Governing equations

Calculations involved the use of four inter-related models, i.e. mass flow, heat transfer, electromagnetic field and discrete phase. Owing to Maxwell's field equations, Ohm's Law and discrete phase model (DPM) equations, source terms were added to momentum and energy equations. These terms account for the contribution of bubbles released from the electrode surface, the electromagnetic field, the flow and heat transfer phenomena.

#### 2.1.1. Magnetohydrodynamic phenomena

Maxwell's equations accounting for Joule heating [18] were initially solved throughout the computational domain (figure 1). In the absence of free charge density, these equations revert to the following form [19]:

2.1 $$\begin{array}{r} \nabla \cdot \text{E}=0, \end{array}$$

2.2 $$\begin{array}{r} \nabla \cdot \text{B}=0, \end{array}$$

2.3 $$\begin{array}{r} \nabla \times \text{E}=-\frac{\vartheta B}{\vartheta t} \end{array}$$

2.4 $$\begin{array}{r} \;\text{and}\;\nabla \times \text{B}=\sigma \mu_{0}\text{E}. \end{array}$$

where **E** is the electric field (V m^−1^), **B** is the magnetic field (T), *σ* is the electrical conductivity (S m^−1^) and μ_0_ is the magnetic permeability (H m^−1^). Ohm's Law for a fluid with a velocity field **u** inside a magnetic field is described by equation (2.5). The first term on the right-hand side of equation (2.6) is due to the applied (external) electric field and the second term is due to the induced field

2.5 j=σ(E+u×B)

and

2.6 $$\text{B}=B_{0}+b,$$

where **j** is the current density (A m^−2^), **B_0_** is the magnetic field equivalent to the external magnetic field (T) and **b** refers to the induced magnetic field (T). All transient computations were carried out with ANSYS Fluent™; however, Fluent™ does not support calculation of the induced magnetic fields, consequently here we only accounted for the applied magnetic field. Continuity for the electric current requires

2.7 ∇⋅j=0.

The electric field can be written as the gradient of an electric potential

2.8 E=−∇φ.

From equations (2.5), (2.7) and (2.8), it may be seen that the electric potential satisfies equation

2.9 $$\nabla^{2}\varphi =\nabla \cdot (\text{u}\times \text{B}).$$

Heat generation, *q*, due to the material's resistance is calculated from the following equation

2.10 $$q=\frac{j\cdot j}{\sigma }.$$

#### 2.1.2. Transport and phase-change phenomena

For the convective motion, the momentum equations corresponding to a fluid of variable density are written as [20,21]

2.11 $$\frac{\vartheta }{\vartheta t}\rho +\nabla \cdot (\rho u)=0$$

and

2.12 $$\frac{\vartheta }{\vartheta \text{t}}\rho \text{u}+\nabla \cdot (\rho \text{uu})=-\nabla \text{P}+\nabla \cdot (\mu \nabla u)+\rho g+\text{j}\times \text{B}+{\text{S}}_{b}+{\text{S}}_{u},$$

where, **u** is the velocity vector field (m s^−1^), *ρ* is the fluid density (kg m^−3^), *P* is the pressure (Pa) and *μ* is the dynamic viscosity (Pa s). **B** and **j** are the magnetic field intensity (T) and the current density vector field (A m^−2^), respectively, which together produce the Lorentz force source term in (2.12). The use of a variable density is required in order to cater for thermal buoyancy effects in the EAF bath.

The momentum transfer between continuous phases and the discrete phase (*S*_b_) is formulated by expressing the change in momentum of a particle as it passes through each control volume. This momentum change is computed as

2.13 $$S_{b}=\sum \left[\frac{18\mu c_{D}Re}{\rho_{p}d_{p}^{2}24}(u-u_{p})+F_{\mathrm{other}}\right]{\dot{m}}_{p}\Delta t,$$

where *μ* is the dynamic viscosity of the fluid (Pa s^−1^), *ρ*_p_ is the particle density (kg m^−3^), *d*_p_ is the diameter of the particle (m), *Re* is the Reynolds number, *c*_D_ is the drag coefficient, *u* is the velocity of the fluid (m s^−1^), *u*_p_ is the velocity of the particles (m s^−1^), ${\dot{m}}_{p}$ is the mass flow rate of the particles, *F*_other_ are other interaction forces and Δ*t* is the time step (s) [22].

The source term *S*_u_ modifies the momentum balance depending on completion of the phase change, by dampening the velocity at the phase-change interface so that it becomes equal to that of the solidified phase after the transition [23]. The source term follows from equation [16,23]

2.14 $$S_{u}=\frac{{\left(1-a\right)}^{2}}{a^{3}+\varepsilon }A_{\mathrm{mush}}(u-u_{\mathrm{solid}}),$$

where *α* represents the volume fraction of the liquid phase, *A*_mush_ and *ε* represent arbitrary constants, respectively, (*A*_mush_ should be large enough and *ε* small enough to yield proper damping) [23] and *u*_solid_ is the velocity of the solidified material (m s^−1^). The temperature field is described by the energy conservation equation [20]

2.15 $$\rho C_{p}\frac{\vartheta T}{\vartheta t}+(\rho C_{p}u\cdot \nabla )\text{T}=\nabla \cdot (k\nabla \text{T})+\frac{\text{j}\cdot \text{j}}{\sigma },$$

where *T* is the temperature (K), *C*_p_ is the heat capacity (J kg^−1^ K^−1^), *k* is the thermal conductivity (W m^−1^ K^−1^) and *σ* is the electrical conductivity (S m^−1^). The last term on the right-hand side of equation (2.15) constitutes the energy source term which is generated by the flow of electric current (Ohmic heating) [24].

#### 2.1.3. Discrete phase model

The bubbles released from the outer surfaces of the electrodes were modelled using the DPM. The trajectory of a discrete phase particle (e.g. droplet or bubble) is computed by integrating the force balance on the particle, written in a Lagrangian reference frame. This force balance equates the particle inertia with the forces acting on the particle, and can be written (for the *x* direction in Cartesian coordinates) as [22]

2.16 $$\frac{\text{d}u_{p}}{\text{d}t}=F_{D}(u-u_{p})+\frac{g_{x}(\rho_{p}-\rho )}{\rho_{p}}+F_{x},$$

where *F_x_* is an additional acceleration (force per unit particle mass) term and *F*_D_(*u* − *u*_p_) is the drag force per unit particle mass expressed as [22,25]

2.17 $$F_{D}=\frac{18\mu }{\rho_{p}d_{p}^{2}}\;\frac{c_{D}Re}{24}.$$

Here, *u* is the fluid phase velocity (m s^−1^), *u*_p_ is the particle velocity (m s^−1^), *μ* is the molecular viscosity of the fluid (Pa s^−1^), *ρ* is the fluid density (kg m^−3^), *ρ*_p_ is the density of the particle (kg m^−3^) and *d*_p_ is the particle diameter (m). *Re* is the relative Reynolds number, defined as [22,26]

2.18 $$Re=\frac{\rho d_{p}|u-u_{p}|}{\mu }.$$

In equation (2.16), *F_x_* is the ‘virtual mass' force, i.e. the force required to accelerate the fluid surrounding the particle, defined as

2.19 $$F_{x}=\frac{1}{2}\;\frac{\rho }{\rho_{p}}\;\frac{\text{d}}{\text{d}t}(u-u_{p}).$$

Finally, the particle temperature is updated according to a heat balance relating the sensible heat change in the particle to the convective and latent heat transfer between the particle and the continuous phase:

2.20 $$m_{p}C_{p}\frac{\text{d}T_{p}}{\text{d}t}=kA_{p}(T_{\infty }-T_{p})+\epsilon_{p}A_{p}\sigma_{SB}(\theta_{R}^{4}-T_{p}^{4}).$$

where *m*_p_ is the particle mass (kg), *C*_p_ is the particle heat capacity (J kg^−1^ K^−1^), *T*_p_ is the droplet temperature (K), *k* is the convective heat-transfer coefficient (W m^−2^ K^−1^), $\;T_{\infty }$ is the temperature of the continuous phase (K), $\epsilon_{p}$ is the particle emissivity (dimensionless), *A*_p_ is the particle surface (m^2^), *σ*_SB_ is the Stefan–Boltzmann constant (5.67 × 10^−8^ W m^−2^ K^−4^) and $\theta_{R}^{4}$ is the radiation temperature (K).

### 2.2. Boundary conditions

The EAF model boundary conditions are shown in figure 1. As was discussed in Model formulation, the firebricks and air regions have been ignored; specific to the air region, our precursor work indicated that low air viscosity invariably results in unacceptably high Courant numbers which cause simulation instability and divergence. Consequently, we determined new boundary conditions as follows: in the upper wall of the slag region (figure 1), a constant temperature of 1449 K with heat-transfer coefficient of 0.01 W m^−2^ K^−1^ was specified. This temperature was 1 K lower than the melting point of the slag layer; consequently, it does not contribute to the phase change of the slag. In the slag sidewalls, a Dirichlet boundary condition with a value of 1041 K was applied. Similarly, along the ferronickel side and bottom walls (figure 1), constant temperature boundary conditions of 1420 and 1489 K, respectively, were applied, as determined by steady-state simulations (inclusive of firebricks).

At the electrode boundaries, a direct current (DC) voltage was applied [27], while a constant electric potential of ±400 V was applied to the electrode edges. On the sidewalls as well as at the bottom of the furnace, a ground potential (0 V) was used. At the upper boundary of the slag layer the normal gradient of the electric potential was assumed to be zero [10,13,17,28]. Owing to the fact that the flow phenomena were solved only within the control volume containing the slag, no-slip wall conditions were specified at the interface walls between the slag and air, electrodes, ferronickel and firebricks [10,12,13,17].

### 2.3. Materials properties

We have considered the air, slag and ferronickel phases as homogeneous fluid continua. The density and viscosity of the slag layer as well as the electrical conductivity of the ferronickel layer were specified as piecewise linear functions of temperature in order to incorporate temperature variability into the models. Moreover, due to the uncertainty involved in the determination of the electrical and thermal conductivity of the slag, trial ranges between 0.5 and 10.0 S m^−1^ and 3.0–7.0 W m^−1^ K^−1^ were used, respectively [8]. Also, slag viscosity in the range 0.002–0.2 kg m^−1^ s^−1^ was deemed adequate for the study of the influence of the Lorentz force on melt velocities. The physical properties of the materials used in the simulations are summarized in table 1.

**Table 1.** Thermophysical properties of materials used in the simulations. The viscosity of the slag has been estimated via use of the Mills & Sridhar model, while infinite viscosity has been assumed in the solid regions [29–36].

properties	slag	ferronickel	electrodes	firebricks
density (kg m^−3^)	3501–0.339*T*	7000	1360	3210
viscosity (kg m^−1^ s^−1^)	879205.17 × exp(−9.06*E*−3*T*)	0.005	—	—
heat capacity (J kg^−1^ K^−1^)	1445	525	1800	1000
thermal conductivity (W m^−1^ K^−1^)	3–7	12–15	8	2.6
electrical conductivity (S m^−1^)	0.5–3	10^6^–330.83*T*	25 000	0.01
solidus temperature (K)	1420	1570	—	—
liquidus temperature (K)	1450	1600	—	—
latent heat (J kg^−1^)	400 000	290 000	—	—

### 2.4. Computational details

**Figure 2.**
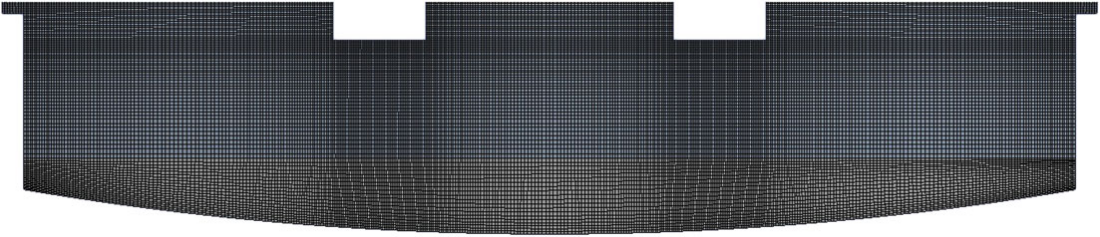
Schematic of the computational grid used in the current study. The figure also illustrates the refined regions around the electrodes.

The computational grid was created using the Mesh application in the ANSYS Workbench™ 15.0 software environment. An example of a coarse grid consisting of 43 669 elements is shown in figure 2. Consistently converging solutions (grid-independence) were obtained for tetrahedral meshes of 129 496 elements with a maximum side length of 0.003. The grid quality indices are presented in table 2. The relaxation factors and discretization methods are listed in table 3. For the velocity and pressure coupling the pressure implicit with splitting of operator (PISO) method was used, due to its effectiveness in solving transient flows [22]. The simulation time step was varied according to the Courant–Friedrichs–Levy stability criterion. The maximum Courant number was set to 0.3, which was observed to be the highest value allowing control of the simulation time step.

**Table 2.** Grid quality indexes.

index	min value	max value	average value	optional value
elements quality	0.7396	0.9994	0.9616	1
aspect ratio	1	2.1710	1.214	1
skewness	3 × 10^−7^	0.1075	9.71 × 10^−3^	<0.8
Jacobian	1	1.0802	1.0007	1

**Table 3.** ANSYS Fluent™ solutions controls for the electric arc furnace model.

relaxation factors		discretization		convergence criteria^a^	
pressure	0.3	pressure	PRESTO!	continuity	10^−4^
density	1	P–U Couple	PISO	velocity	10^−4^
body forces	1	momentum	2nd O-U	energy	10^−7^
momentum	0.7	volume fraction	Geo-Reconstruct	electric potential	10^−9^
energy	1	energy	2nd O-U		
solidification	0.9	electric potential	1st O-U		
electric potential	0.9				
DPM	0.5				

^a^Concerning the maximum permissible value of the respective equations residuals.

## 3. Results and discussion

We will first discuss the results of our sensitivity analysis with regard to the effect of Lorentz forces, CO bubbles, slag and ferronickel properties as well as parameters related to the electrodes geometries; this analysis was aimed at the determination of the basic flow and heat-transfer mechanisms which are likely to occur during melting.

### 3.1. Electromagnetic (Lorentz) forces calculation

In our investigation of the effect of Lorentz forces on bath stirring, we note that in the systems studied temperatures may exceed the Curie temperature, over which the ferromagnetic material becomes paramagnetic. In our current magnetohydrodynamics (MHD) formulation, the induced magnetic field was modelled as an external field, the values of which were taken to be 10 to 100 times higher than those in the relevant literature [15,37,38], in order to test whether the effect of the magnetic field may be ignored. As indicated by the data in table 4, the magnetic field contribution is, in fact, negligible, in accordance with our previous three-dimensional modelling of the EAF [9]. Lorentz forces become appreciable for slag conductivities higher than 2000 S m^−1^.

**Table 4.** Correlation between applied magnetic field and Lorentz forces and velocities created in the bath.

**B** (T)	slag viscosity (kg m^−1^ s^−1^)	ferronickel viscosity (kg m^−1^ s^−1^)	Lorentz force (N m^−3^)	max velocity (m s^−1^)	mean velocity (m s^−1^)
3	0.2	10^−4^	46–1.77 × 10^4^	0.21	6.9 × 10^−3^
0.3	0.2	10^−4^	6.8–1.36 × 10^2^	0.08	1.8 × 10^−3^
3	0.002	10^−4^	25–2.59 × 10^4^	0.26	8 × 10^−3^
0.3	0.002	10^−4^	7.86–1.57 × 10^2^	0.097	3.2 × 10^−3^

### 3.2. Heat-transfer and phase-change phenomena

**Figure 3.**
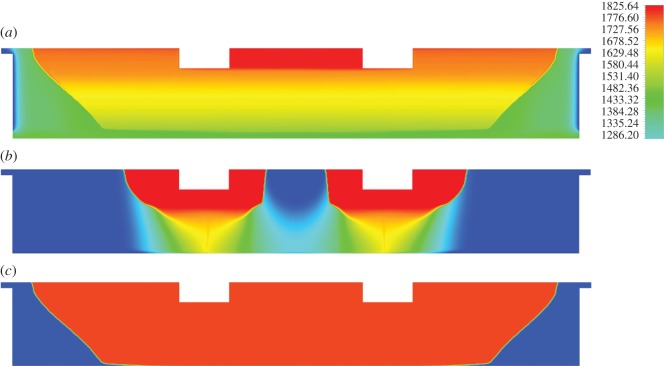
(*a*) Temperature distribution [K] in the electric submerged arc furnace (*b*) and (*c*) distribution of liquid fraction in the slag region with respect to the time.

The temperature and liquid fraction distributions in the EAF at two different time steps are presented in figure 3*a,c*, respectively. In the temperature profile, several distinct zones may be observed. Principally, the region between the two electrodes is the locus of the highest computed temperatures (approx. 1800 K), the latter gradually decreasing towards the slag–ferronickel interface. The regions adjacent to the slag layer sidewalls as well as the ferronickel layer exhibit temperatures about 200–300 K lower than the values computed in the vicinity of the electrodes. Finally, the sidewall surfaces had the lowest temperatures (1050–1100 K), owing to substantial heat transfer to the coolant; this region remained in the solid phase throughout the process (solidified zone). All computed temperatures were also confirmed experimentally. Measurements of the slag temperature by LARCO S.A. via infrared pyrometer, indicated that temperature varied between 1523 and 1633 K at the outlet of the EAF. Also, near the electrodes the temperature varied between 2273 and 3073 K.

Figure 3*b,c* depict snapshots of the melting process at two different time steps. It is evident that the melting phenomenon initially occurs in the vicinity of the two electrodes due to intense Joule heat. The melting profile first progresses along the EAF vertical axis and then spreads radially towards the sidewalls. As may be inferred from figure 3*a*, no melting occurs at the regions immediately adjacent to the sidewalls due to insufficient heating.

### 3.3. The development of velocity field

**Figure 4.**
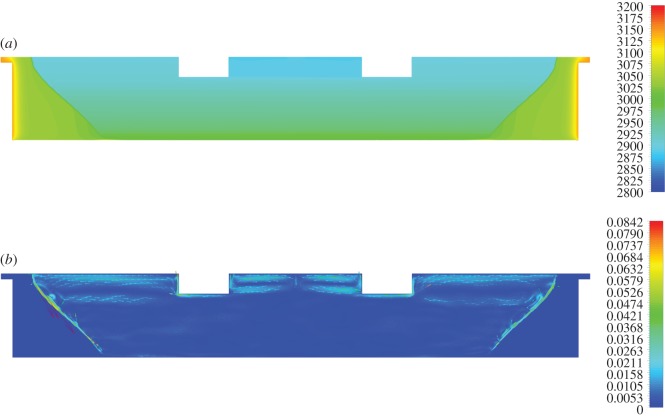
(*a*) Density (kg m^−3^) distribution in the slag region. (*b*) Velocity magnitude contours (m s^−1^) with velocity magnitude vectors in the slag region.

The density profile within the slag bath is shown in figure 4*a*. Assuming that density is temperature-dependent, the results are fully consistent with the trend in figure 3*a*; regions with high temperature exhibit the lowest density and vice versa. These results are also in accordance with velocity vector field computations, depicted in figure 4*b*. The main stirring phenomenon in the bath is related to thermal buoyancy due to density differences in the various regions. Upon transition to the melt phase, the liquid tends to move upwards, thus creating a buoyancy effect. It can be observed that several vortices are also present in the bath (e.g. figure 4*b*): two large eddies in the electrode sidewall area and two smaller ones between electrodes. The direction of these vortices may be explained on the basis of buoyancy-driven fluid motion.

**Figure 5.**
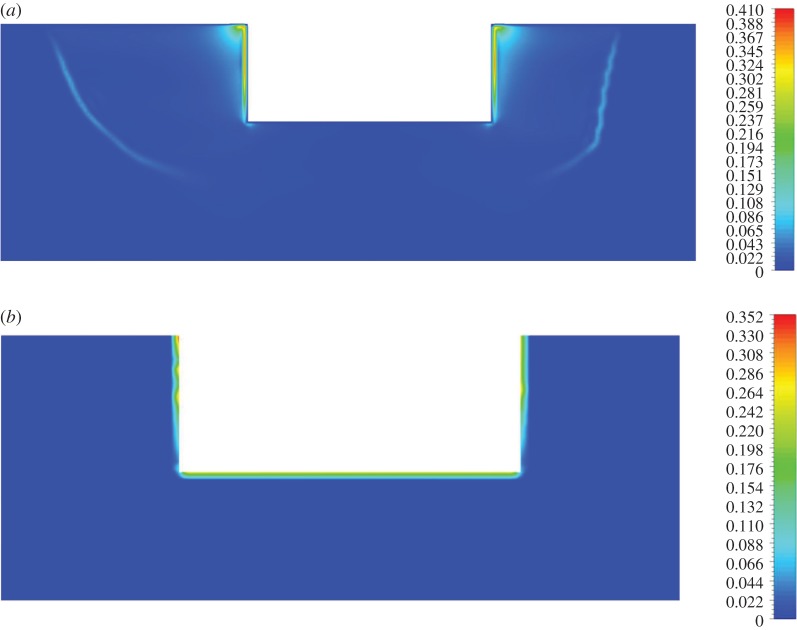
(*a*) Velocity distribution (m s^−1^) near the electrodes due to the bubbles release. (*b*) Bubbles mass (kg m^−3^) concentration near the electrodes region.

Figure 5*a,b* portray the velocity distribution inclusive of the emission of CO bubbles from electrode sidewalls; the maximum velocities were generally higher in comparison to the values computed in the absence of CO bubbles (figure 4*b*). However, this phenomenon is essentially restricted to within a small area around the electrodes and quickly faints with increasing distance from the electrode surface. Furthermore, CO concentration in the bath is markedly higher on the electrode sidewalls in comparison to the metal bath, due to the large density difference between slag and CO, resulting in the accelerated escape of the CO bubbles from the upper EAF compartment. We, therefore, consider that it is plausible that the momentum of the CO bubbles does not significantly affect stirring of the metal bath.

### 3.4. The effect of slag properties and electrode geometry

**Figure 6.**
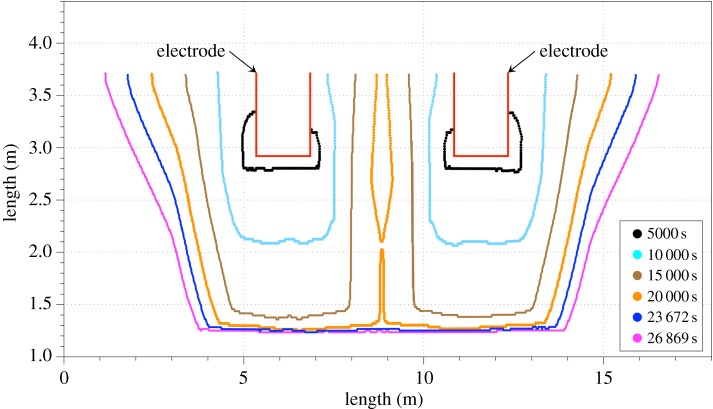
Liquid fraction profile in the slag region with respect to the time (s).

Our transient approach examined the influence of the slag's thermal and electrical conductivity on Joule heat, temperatures and liquid profile distributions as well as on velocity gradients in the bath. Figure 6 shows the liquid fraction profile evolution with respect to time. In this case the immersion depth of the electrodes was set to 0.8 m and the slag thermal and electrical conductivities were 7 W m^−1^ K^−1^ and 6 S m^−1^, respectively. In the first two time steps displayed (5000 s and 10 000 s) it can be seen that melting initially progresses along the vertical axis, followed by spreading of the liquid fraction towards the sidewalls.

**Figure 7.**
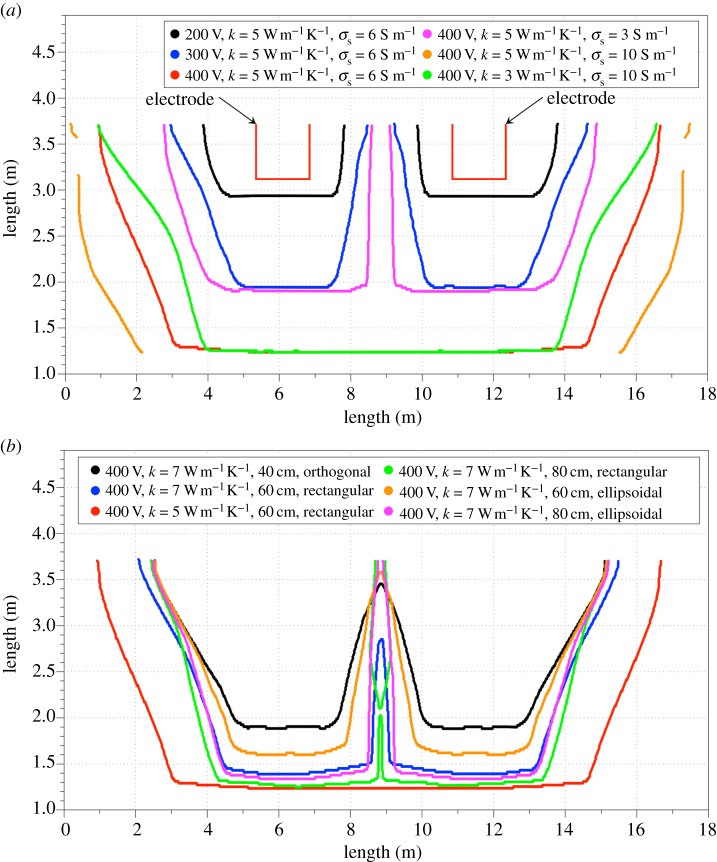
(*a*) Slag region liquid fraction profile with respect to slag's thermal and electrical conductivity. (*b*) Slag region liquid fraction profile with respect to the slag's thermal conductivity, electrodes shape and immersion depth.

Figure 7*a* shows the change in the profile of the liquid fraction of the slag with respect to the electric potential applied to the electrodes, as a function of slag thermal and electrical conductivities. We note that melting is favoured by increasing electric potential and slag electrical conductivity. These findings are potentially important to the fine tuning of the industrial EAF operation as modifying the Si, Al and Fe slag content, among others, may severely impact its electrical conductivity [8].

For sufficiently high values of its electrical conductivity (e.g. 10 S m^−1^), melting is favoured by the increase of the thermal conductivity of the slag. The slag liquid fraction profiles computed for a range of constant slag electrical conductivities (figure 7*b*) reaffirmed that melting is favoured by smaller values of slag's thermal conductivity, as well as by the increase of the electrode immersion depth and the use of rectangular tips. The latter is due to the increased Joule heat on electrode tip corners, as also indicated in our precursor steady-state simulations [7].

### 3.5. Mean and maximum values of the investigated quantities

All simulations parameters used in the current study inclusive of applied electric potential, electrode shape, immersion depth and slag thermal and electrical conductivities are listed in table 5.

**Table 5.** Parameter values considered in the present work.

case	electric potential (V)	electrode type	immersion depth (m)	thermal conductivity (W m^−1^ K^−1^)	electrical conductivity (S m^−1^)
case 1	200	rectangular	60	5	6
case 2	300	rectangular	60	5	6
case 3	400	rectangular	60	5	6
case 4	400	rectangular	60	5	3
case 5	400	rectangular	60	5	10
case 6	400	rectangular	60	3	10
case 7	400	rectangular	40	7	6
case 8	400	rectangular	60	7	6
case 9	400	rectangular	80	7	6
case 10	400	ellipsoidal	60	7	6
case 11	400	ellipsoidal	80	7	6

The mean and maximum velocities computed in the slag region are presented in table 6. The maximum velocities were invariably observed in the vicinity of the electrodes as well as along the slag solid–liquid interface (figure 4*b*). Maximum velocities ranged between 0.028 and 0.130 m s^−1^, in agreement with literature values [6,10–12,37,39–42]. Mean velocities in the slag region varied between 1.84 × 10^−4^ and 4.87 × 10^−3^ m s^−1^, also in alignment with published values [6,10,40,41,43,44]. The data in table 6 indicate that both increasing electrode potential and increasing slag electrical conductivity lead to higher velocities. For smaller slag electrical conductivity (6 S m^−1^), higher velocities are attained for decreasing slag thermal conductivity. The use of rectangular electrode tips leads to higher velocities owing to increased Joule heat in comparison to ellipsoidal tips, which in turn creates more pronounced density variations and consequently more intense buoyancy effects.

**Table 6.** Correlation between the electric potential, shape and immersion depth of the electrodes with the thermal and electrical conductivity of the slag and the associated velocities and dimensionless quantities.

case	max velocity (m s^−1^)	mean velocity (m s^−1^)	average Péclet	max Péclet	average Prandtl	max Prandtl	average Reynolds	max Reynolds
case 1	0.02828	0.00018	5.26	524.8	57.8	57.8	0.09	12.5
case 2	0.08063	0.00109	24.53	1001.8	57.4	57.8	0.42	17.3
case 3	0.10101	0.00282	48.02	1458.2	57.6	57.8	0.83	25.2
case 4	0.05901	0.00090	6.43	524.7	57.6	57.8	0.11	9.1
case 5	0.09464	0.00480	67.06	1234.4	57.6	57.8	1.16	21.3
case 6	0.09757	0.00312	75.51	1829.7	96.0	96.3	0.78	19.0
case 7	0.06526	0.00117	15.45	657.2	41.1	41.3	0.37	15.9
case 8	0.09628	0.00189	23.83	826.3	41.1	41.2	0.57	20.0
case 9	0.13204	0.00488	57.8	3122.2	41.1	41.3	1.41	75.7
case 10	0.06656	0.00107	17.61	483.89	41.1	41.2	0.42	12.0
case 11	0.07379	0.00156	6.14	417.1	41.1	41.3	0.15	10.1

In the melt phase, the relative significance of advective and diffusive mechanisms is identified by the Péclet number (defined as the ratio advective transport rate/diffusive transport rate). Owing to the complexity of the EAF geometry, the current study has considered cell-averaged Péclet numbers. The high Péclet values computed (table 6) suggest that mass transport by advection predominates over diffusion and indicates that bath stirring (a purely advective phenomenon) plays a significant role in the heat transfer and in the flow mechanisms within the bath. The validity of the assumption of laminar flow employed in the current model was confirmed by the resulting low Reynolds numbers (table 6).

Parameter correlations were inferred on the basis of their computed mean and maximum values, as listed in table 7. It is evident that the use of ellipsoidal electrodes leads to lower Joule heat in comparison to rectangular tips. Moreover, the Joule heat produced is favoured by the increase of the electrical conductivity of the slag; the maximum heat value was determined to be 7.43 × 10^6^ W m^−3^ [14,28,41,45], corresponding to a maximum temperature of 2173.3 K.

**Table 7.** Mean and maximum computed values of the parameters studied.

case	max slag temp. (K)	mean slag temp. (K)	max slag Joule heat (W m^−3^)	mean slag Joule heat (W m^−3^)	max FeNi Joule heat (W m^−3^)	mean FeNi Joule heat (W m^−3^)
case 1	1502.4	1410.2	1120303.3	25489.8	20528.5	3.24
case 2	1670.4	1520.3	2518792.1	57357.8	48078.7	7.23
case 3	1825.6	1605.9	4435311.4	101815.0	61125.6	13.47
case 4	1608.4	1452.4	2217769.7	50901.6	84467.0	21.07
case 5	2120.4	1808.1	7385625.3	169673.3	38771.9	19.30
case 6	2173.3	1686.0	7430762.1	169676.2	90967.3	50.54
case 7	1796.1	1375.5	4573024.0	89342.0	76694.2	10.22
case 8	1774.8	1409.7	4475257.3	101925.3	87206.5	12.78
case 9	1756.1	1453.3	4464280.0	115916.2	120432.3	16.26
case 10	1797.3	1393.5	1444542.4	95597.3	73278.5	34.39
case 11	1795.0	1433.0	1408522.0	108888.7	89097.2	42.84

Increasing electrode immersion depth led, on the one hand, to lower Joule heat values while at the same time yielding higher mean Joule heat values in the slag. Also, higher Joule heat was observed in the ferronickel region, due to a higher charge flow flagged by a high current density inside this region, eventually causing it to overheat [27,28,46–48].

Decreasing slag thermal conductivities translated to higher initial temperatures in the slag, followed by increased temperatures in the ferronickel region. As it can be seen in table 1, the electrical conductivity of the ferronickel layer is temperature dependent. Consequently, higher temperatures lead to decreased ferronickel electrical conductivity, which in turn leads to lower Joule heat produced in this region and a significantly lower melting rate.

### 3.6. COMSOL and Fluent MHD comparison

**Figure 8.**
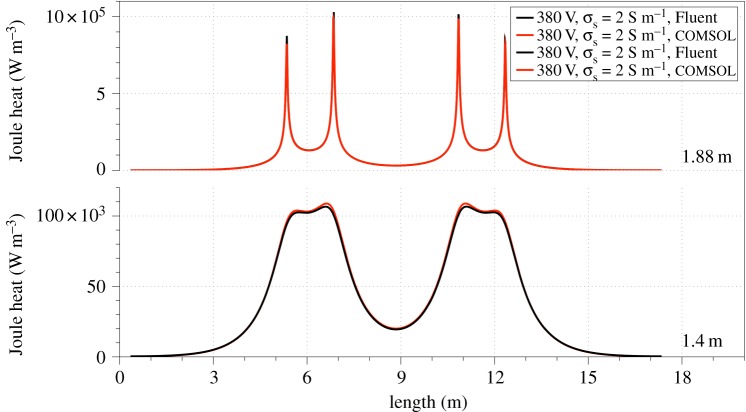
Comparison between the solutions obtained from the COMSOL and Fluent codes.

Finally, we compared the results obtained between the ANSYS Fluent™ v. 15.0 and COMSOL Multiphysics™. In order to compare the results side by side, two horizontal sampling lines were drawn. The horizontal lines stretched from *x*_1_ = 0 m to *x*_2_ = 17 m with constant *y* values of 1.4 m and 1.88 m, respectively (see set-up in figure 1). The Joule heat (W m^−3^) profile along the two horizontal axes is depicted in figure 8. No significant variations were observed in the MHD solutions, between the two solvers.

## 4. Conclusion

A number of consistent conclusions can be drawn:

(1) The electric conductivity of slag and ferronickel has a substantial effect on the Joule heat produced in the furnace and the maximum temperature of the bath. More specifically, it was found that, for values of slag electric conductivity close to 6 S m^−1^, the range of maximum temperatures due to the melting in the furnace was between 1756 and 1825 K. Furthermore, the results show that lower values of slag thermal conductivity lead to higher temperatures in the slag and ferronickel layers. This results in lower electrical conductivities (electrical conductivity is considered to be temperature dependent) and lower Joule heat values in the ferronickel layer.
(2) The contribution of Lorentz forces to bath stirring is negligible in comparison to thermal buoyancy effects. Inclusion of the effect of CO bubbles led to the formation of slag velocities of the order of 0.41318 m s^−1^, compared with 0.02828–0.132044 m s^−1^, which is a typical velocity range computed in the absence of CO bubbles. However, the bubble contribution was found to be strictly localized around the electrodes.
(3) Melting of the slag layer initially occurred in the close proximity of the vertical axis of the EAF, gradually expanding radially towards the furnace walls and the slag–ferronickel interface. Melting was favoured by increasing electric potentials and slag electric conductivities. For high electric conductivity values of slag (10 S m^−1^) the melting process was favoured by an increase of electrical conductivity values. Increasing electrode immersion depth promoted melting. The use of rectangular versus elliptical electrode tips led to an increase of the volume fraction of the molten slag, due to the increased values of Joule heat on electrode edges. Use of rectangular electrode tips also caused the formation of higher velocities within the slag region.
(4) Although increasing electrode immersion depths led to a reduction of the Joule heat within the slag, it also resulted in an increase of the mean Joule heat in this region and an increase of the maximum Joule heat in the ferronickel region, owing to high current densities.
(5) A side-by-side comparison of the ANSYS Fluent™ and COMSOL Multiphysics™ computational codes, showed no significant differences in terms of the electromagnetic models and affirmed the validity of our results.

## Data accessibility

Data available from the Dryad Digital Repository: http://dx.doi.org/10.5061/dryad.bc434 [49].
